# Simultaneous mutations of *LAMB2* and *NPHP1*genes in a Chinese girl with isolated congenital nephrotic syndrome: a case report

**DOI:** 10.1186/s12887-016-0583-0

**Published:** 2016-03-22

**Authors:** Liru Qiu, Jianhua Zhou

**Affiliations:** Department of Pediatrics, Tongji Hospital, Tongji Medical College, Huazhong University of Science & Technology, Wuhan, 430030 China

**Keywords:** Congenital nephrotic syndrome, Pierson syndrome, Juvenile nephronophthisis, Next generation sequencing, Gene mutation

## Abstract

**Background:**

*LAMB2* mutations cause Pierson syndrome (OMIM 609049), an autosomal recessive genetic disease typically characterized by congenital nephrotic syndrome (CNS) and early onset renal failure, as well as bilateral microcoria. *NPHP1* mutations cause familial juvenile nephronophthisis type 1 (*NPHP1*, OMIM 256100), another autosomal recessive renal disease that usually occurs years after birth. Both Pierson syndrome and nephronophthisis cause end-stage renal disease and rare kidney diseases in children. We report an extremely rare case of concurrent mutations of *LAMB2* and *NPHP1* in a Chinese girl with isolated CNS and the association of the phenotype with novel non-truncating mutations of *LAMB2*.

**Case presentation:**

A-34-day-old girl presented with CNS but no eye abnormalities, and mild hyperechogenicity of kidneys. A novel c.1176_1178delTCT mutation caused deletion of a glycine in exon 9 of *LAMB2*, and another mutation c.4923 + 2 T > G led to a splicing error. In addition, compound heterozygous mutations of *NPHP1* were identified in this child using next generation sequencing, and confirmed by Sanger sequencing.

**Conclusion:**

Mutations of the *LAMB2* and *NPHP1* are present in infants with isolated CNS. Next generation sequencing enabled high-throughput screening for mutant genes promptly, with clinically significant outcomes. In addition, our results expand the phenotype spectrum of *LAMB2* mutations as the only renal manifestation.

## Background

*LAMB2* (MIM# 150325) mutation typically causes Pierson syndrome (OMIM 609049), an autosomal recessive disease presenting as congenital nephrotic syndrome (CNS) and early onset renal failure, as well as bilateral microcoria. Most mutations causing Pierson syndrome lead to truncation of the laminin β2 chain, and thus seriously impair the glomerular basement membrane. The phenotypes of non-truncating mutations range from a mild form of Pierson syndrome to isolated CNS [1, 2].

*NPHP1* (MIM# 607100) mutation causes familial juvenile nephronophthisis type 1, another autosomal recessive disease manifesting as medullary cysts and chronic renal failure, which usually occurs in the first decade of life [3].

Both Pierson syndrome and familial juvenile nephronophthisis cause end-stage renal failure and are rare in children [1, 4]. Pierson syndrome is especially rare, and has been reported in no more than 50 families including one Chinese family. Concurrent mutations of *LAMB2* and *NPHP1* are not anticipated, because of their location on chromosomes 2 and 3, respectively. Here we report an extremely rare case of co-existing mutations in a Chinese girl with isolated CNS and find that the phenotype in this case may be associated with novel non-truncating mutations of the *LAMB2* gene.

## Case presentation

The patient is the first child of non-consanguineous Chinese parents. She was born by cesarean delivery after 36 weeks and 3 days of gestation since her mother suffered from appendicitis. The pregnancy was unremarkable, and routine prenatal ultrasound evaluation was performed. No positive family history of disease was found. Her birth weight was 2.7 kg. The placental weight and amniotic fluid volume were normal. After birth, she was sent to NICU for mild respiratory distress, which was thought to be due to neonatal pneumonia. Edema in her legs was noted, but unfortunately urine analysis was not performed.

The girl was first admitted to our hospital at 34 days after birth due to worsening edema. Physical examination showed her bodyweight was 5.0 kg (>97th) and her body length was 50 cm (50th). Mild ascites and edema in the face, legs, and periorbital area was noted, and no ocular anomalies were found. Laboratory data revealed a total serum protein level of 25.7 g/L, albumin 16.1 g/L, total cholesterol 2.72 mmol/L, and serum creatinine 20 μmol/L. Her electrolytes were within the normal range except for mild hyperkalemia. Serological tests were negative for syphilis, toxoplasmosis, rubella, cytomegalovirus (CMV), hepatitis B virus, hepatitis C virus, herpes virus types 1 and 2, and HIV. Urine analysis showed proteinuria of 3+ and hematuria of 3+. The urinary albumin-to-creatinine ratio was 53.5 mg/mg. Renal ultrasonography showed the size of the right kidney as 61 × 26 mm (mean of right renal size in a Chinese infant aged 30 days: 54.4 ± 3.5 × 31.4 ± 2.7 mm [5]) and that of the left kidney at 57 × 26 mm (mean of left renal size in a Chinese infant aged 30 days: 54.2 ± 3.5 × 31.4 ± 2.9 mm [5]) with mild hyperechogenicity and collecting system separation (the right was 3 mm and the left was 5 mm).

Ophthalmic examination did not indicate strabismus, retinopathy, or microcoria.

Urinalysis and renal ultrasound results of her parents were negative.

Renal biopsy was not performed.

The child was diagnosed with CNS. Intravenous albumin and diuretics were administered. She was treated with hydrochlorothiazide, anti-sterone and fosinopril. However, facial and lower limb edema persisted. Her parents decided to discontinue the treatment and refused to return to the hospital until she had fever and cough three months later. She showed serious edema and dyspnea but still no ocular abnormalities. Finally she died of renal failure and respiratory infection.

### Gene mutation analysis

In order to investigate the underlying genetic defects for congenital nephrotic syndrome and renal failure in this case, we performed next generation sequencing of a panel of 21 genes (*ACTN4, ALG1, APOL1, CD2AP, COQ2, INF2, LAMB2, LMX1B, MYO1E, NPHS1, NPHS2, PDSS2, PLCE1, PMM2, PTPRO, SCARB2, SMARCAL1, TRPC6, WT1, ZMPSTE24,* and *NPHP1*) underlying the most common genetic nephrotic syndrome or renal failure when the girl was first admitted to our hospital. Briefly, genomic DNA of the child and her parent was extracted from peripheral blood with QIAamp DNA Mini Kit (Qiagen, Germany). All exons of the 21 genes listed above were captured with a microarray chip and all exons with the flanking 10 bp introns were sequenced with Illumina HiSeq2000. Image analysis and base calling were performed using the Illumina Pipeline. To identify single-nucleotide variants (SNVs) and indels, we aligned the 90 bp clean reads against the human genome reference from the NCBI database (HG19) using the BWA (Burrows Wheeler Aligner) Multi-Vision software package. SNVs and indels were identified using SOAPsnp software and Samtools, respectively. The average sequencing depth for target region was more than 449-fold.

**Fig. 1:**
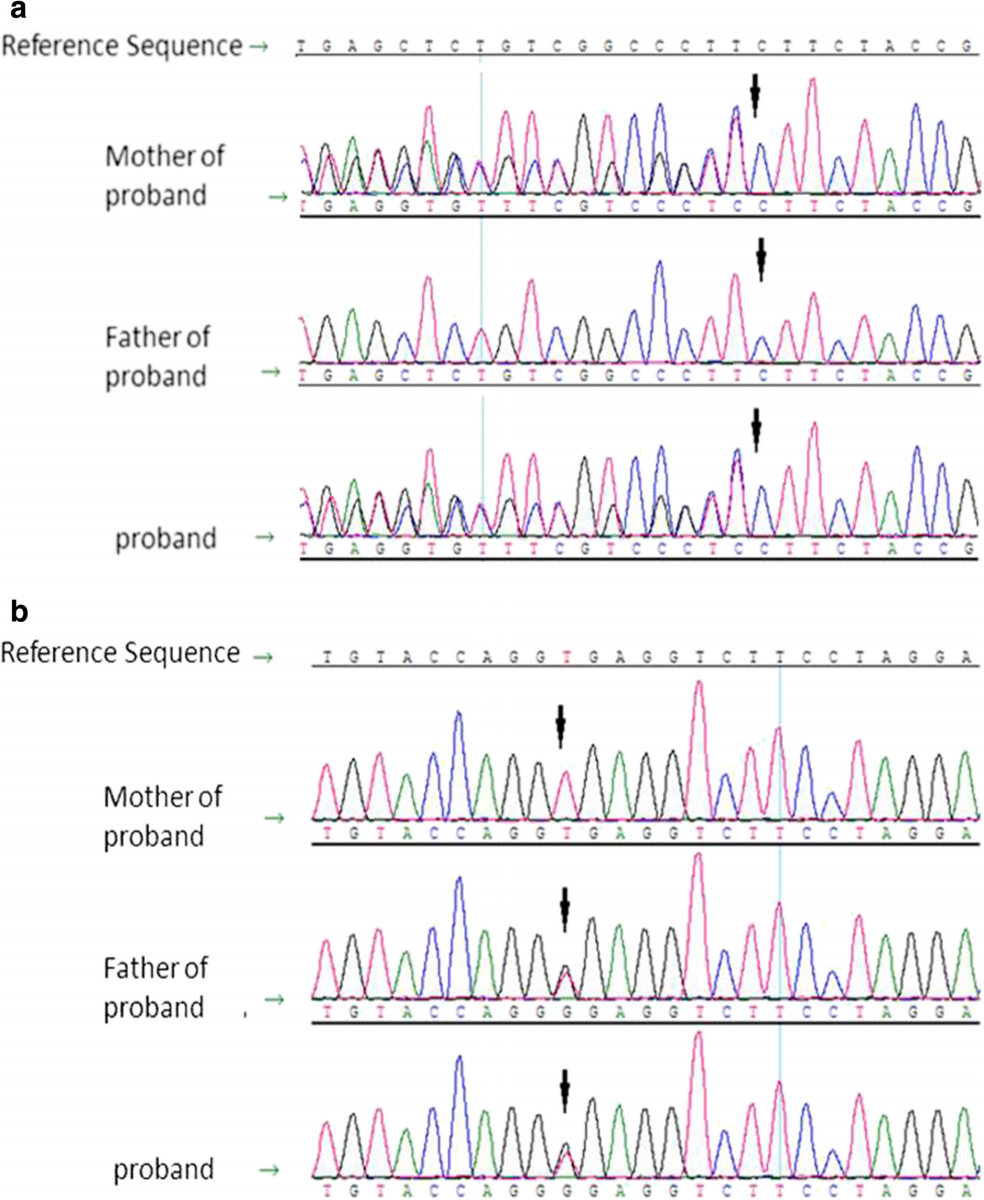
Sequencing of the *LAMB2* gene. **a** DNA sequencing profile showing exon9 c.1176_1178delTCT mutation in *LAMB2,* which was derived from the patient’s mother. The arrow indicates the position of the mutation. **b** The intron 29 c.4923 + 2 T > G mutation in *LAMB2* was inherited from the patient’s father. The arrow indicates the position of the mutation

As shown in Table 1, two novel mutations of *LAMB2* were identified in the child*.* The c.1176_1178delTCT resulted in deletion of a glycine in exon 9 of laminin β2. The other mutation c.4923 + 2 T > G was located in the splice site of intron 29, leading to a splicing error. These two mutations were verified in her parents by direct sequencing of PCR-amplified products of exon 9 and intron 29, including the splice site with the Sanger method (Fig. 1a and b). The first mutation was inherited maternally, and the second one paternally. Her parents were healthy carriers of *LAMB2* gene mutations.

**Table 1:** *LAMB2* and *NPHP1* mutations

Gene	region	Nucleotide change	Reference transcript	Amino acid change	Chromosomal location	Hom /Het*
*LAMB2*	Exon9	c.1176_1178delTCT	NM_002292.3	p. Phe392del	Chr3:49167708	Het
*LAMB2*	Intron29	c.4923 + 2 T > G		-	Chr3:49159375	Het
*NPHP1*	Exon 8	c.922 T > C	NM_000272.3	p.Ser308Pro	Chr2:110922114	Het
*NPHP1*	Exon 17	c.1757G > A		p.Arg586Gln	Chr2:110889309	Het

*Hom: homozygote, Het: heterozygote

**Fig. 2:**
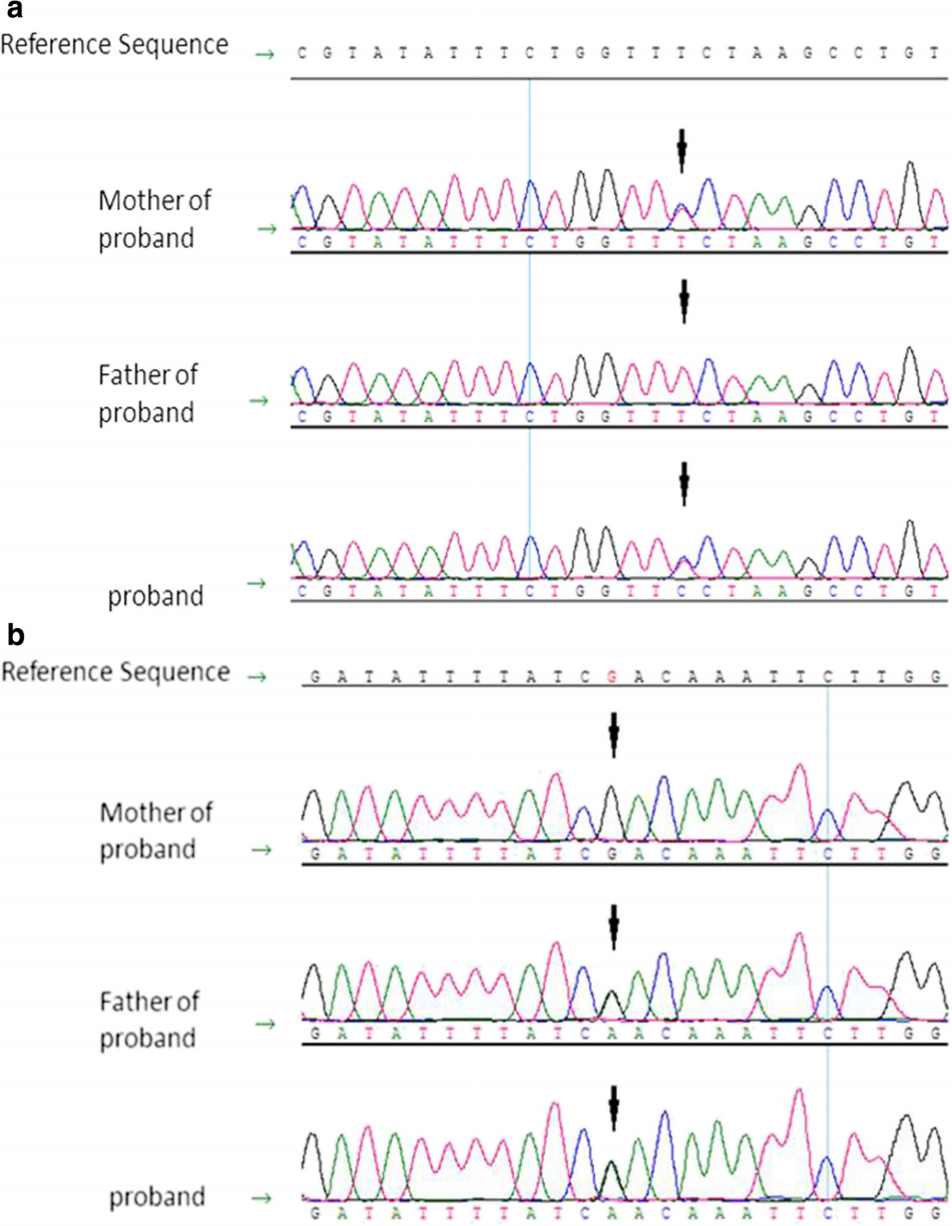
Sequencing of the *NPHP1* gene. **a** DNA sequencing profile showing exon8 c.922 T > C (p.Ser308Pro) in *NPHP1,* which was derived from the patient’s mother. The arrow indicates the position of the mutation. **b** The exon 17 c.1757G > A (p.Arg586Gln) mutation in *NPHP1* was inherited from the patient’s father. The arrow indicates the position of the mutation

Unexpectedly, compound heterozygous mutations of the *NPHP1* were simultaneously found in this child. The maternal mutation of 922 T > C in exon 8 led to a substitution of serine with proline, and the paternal mutation of 1757G > A in exon 17 caused a substitution of arginine with glutamine (Table 1). These two mutations in the *NPHP1* gene were also verified in her family by direct sequencing of PCR-amplified products of exon 8 and exon 17 with the Sanger method (Fig. 2a and b). Her parents were also healthy carriers of *NPHP1* mutations.

Primers used for *LAMB2* and *NPHP1* are shown in Table 2.

**Table 2:** Primers used for *LAMB2* and *NPHP1*

Gene	Primer name	Sequence (5'-3')	Size (bp)
*LAMB2*	Primer 9F	CTGGCATCTGGCAATGTGAGTG	390
	Primer 9R	AGCGACCACCGTCTTGAGAAC	
	Primer 29F	TACAGGCAGCACTGGAGGA	441
	Primer 29R	GGTTAGGTTAGCGTGGTAGC	
*NPHP1*	Primer 8F	AATAGGATGGAGACTGTGGAAGA	560
	Primer 8R	CCTGTCACTCATGCTCTGGA	
	Primer17F	TCAGGACTTATTCCAGGTCTGTCA	587
	Primer17R	GCACATAAGGAAGTGGCAAAGC	

None of the above mutations in the *LAMB2* and *NPHP1* was found in 100 healthy Chinese controls or in the 1000 human genome database. All four mutations were assumed detrimental based on analyses with both SIFT and PolyPhen software. The substitution of arginine with glutamine caused by the mutation of 1757G > A in exon 17 of *NPHP1* was present in the ExAC database and was confirmed as the pathogenic variant.

No mutation was found in the other 19 genes following next generation sequencing.

## Discussion

CNS is defined as nephrotic syndrome occurring within the first 3 months of life [6]. CNS is clinically and genetically heterogeneous. Perinatal infections, especially syphilis, CMV, and HIV infection as well as toxoplasmosis, were reported as the causes of CNS in a small proportion of patients [7–9]. The patient in the case described here was repeatedly examined for syphilis, toxoplasmosis, rubella, HIV, CMV, and other pathogens. There was no evidence of infection. Therefore, next generation sequencing was used to screen mutations in 21 genes responsible for the most common genetic nephrotic syndrome or renal failure. Surprisingly, we found concurrent mutations in both *LAMB2* and *NPHP1* genes, for the first time in a child with congenital nephrotic syndrome.

Mutations of the *LAMB2* gene, which is located on chromosome 3, typically cause Pierson syndrome [1]. *LAMB2* is comprised of 32 exons and encodes laminin β2, a protein with 1798 amino acids. *LAMB2* mutations were assumed to be detrimental in this case using both SIFT and PolyPhen software analyses. We considered these mutations as probably pathogenic, although functional analysis is needed. Laminin is a family of heterotrimeric extracellular glycoproteins that are composed of α-, β-, and γ-chains. Laminin β2 is the major non-collagenous component of basement membrane and is expressed in mature glomeruli, retina, iris muscle, and lens capsule. Genetic defects of the laminin β2 chain may alter the normal structure of the glomerular basement membrane and impair the integrity of the glomerular filtration barrier [10]. Similarly a laminin β2 chain deficiency results in aplasia or hypoplasia of the dilator muscles of the iris. Therefore, patients with Pierson syndrome typically present with not only CNS or nephrotic syndrome in infancy or even later, but also with microcoria, which is a relatively constant feature and is usually the first noted sign of Pierson syndrome. Interestingly, the patient in our case displayed an uncommon phenotype in that she developed CNS with early renal failure but showed no microcoria and other anomalies such as strabismus or retinopathy upon ophthalmic examination. Due to early renal failure and death at 4 months, we were unable to observe other ocular anomalies and medullary cysts.

To date, a total of only 46 unrelated families including 98 patients with Pierson syndrome have been reported [2, 11–17]. All except five patients have shown ocular anomalies with a wide spectrum of clinical conditions, three patients were not examined and the other two patients showed no such anomalies. A few patients initially presented with only minor ocular malformations or even without ocular malformations, but they still had a high risk of developing serious ocular complications. Mohney [14] identified a novel mutation of *LAMB2* (c. 440A > G; His147R) in a multi-generational consanguineous family with 52 members. All of the affected living members, ranging in age from 3 to 42 years, had ocular abnormalities such as pigmentary retinopathy, and unilateral or bilateral retinal detachment. None of the 52 family members had microcoria. Therefore, microcoria is not a defining characteristic of *LAMB2* mutation*-*associated disorders.

Kidney involvement is thought to be an invariant manifestation of defects of *LAMB2* regardless of the age at onset. The predominant age of onset of nephrotic syndrome was reported to be within 3 months (in 79/98) [2, 11–17]. Most patients displayed progressive decline in renal function and early onset of end-stage renal disease, although several cases still retained normal renal function at an elderly age. The members of the aforementioned multi-generational consanguineous family [14] showed various kidney manifestations. A few cases required transplantation at younger age, but one 37-year-old patient had a stable glomerular filtration rate of 53 ml/min/1.73 m^2^. These results, together with our case, strongly indicate that the spectrum of renal phenotype varies widely in *LAMB2* gene-mutated cases.

Until now, only four patients with Pierson syndrome have been reported from Asia [2, 11, 12]. Two patients were in Korea [11], one with truncating mutations and the other with a missense mutation in one allele and a frame-shift deletion in another allele. A Chinese patient with truncating mutations developed nephrotic syndrome at 3 years of age [2], and a Japanese patient had compound heterozygous mutations [12]. Two of these patients developed end-stage renal failure during infancy. One patient had a normal range GFR at 3 years of age. Data from the fourth patient were not available, but it is known that the patient died at age 7. In contrast, our case showed novel non-truncating compound heterozygous mutations of the *LAMB2* gene associated with CNS and renal failure.

Nephronophthisis (NPHP) is an autosomal recessive disease of kidney cysts and one of the leading genetic causes of end-stage renal disease during the first three decades of life [4]. Mutations of the *NPHP1* gene, which is located on chromosome 2, account for the underlying genetic defects in approximately 21 % of all patients with NPHP. *NPHP1* encodes nephrocystin-1 that is expressed in cell-cell junctions of collecting duct cells. To date, more than 13 genes have been implicated in this disease [18]. Patients with *NPHP1* mutations resulting in end-stage renal disease were aged more than 6 years with a mean age of 12.5 years. Gene mutation analysis of our case identified the compound heterozygous missense mutations of c.922 T > C (p.Ser308Pro) in one allele, which is a novel mutation and c.1757G > A (p.Arg586Gln) present in the ExAC database of the other allele of *NPHP1*. These mutations, which result in substitution of highly-conserved serine and arginine with proline and glutamine, respectively, are predicted to be pathogenic by both SIFT and Polyphen software analysis. The patient in our case did not develop corticomedullary cysts in the kidneys and experienced renal failure soon after birth, suggesting that the *LAMB2* mutations but not the *NPHP1* mutations played a decisive role in renal failure in this case. No typical manifestations of nephronophthisis were observed in our case since she passed away before the age of onset of nephronophthisis. We assumed that the *NPHP1* mutation was pathogenic because a variant was previously reported to be pathogenic in ExAC database. The other mutation was not found in 100 healthy Chinese controls and in the 1000 human genome database and was predicted to be detrimental through both SIFT and PolyPhen software analyses.

Next generation sequencing is a powerful technique for high-throughput screening of gene mutations cost-effectively and rapidly. The identification of several novel gene variants is a great diagnostic challenge. In addition to functional verification of new gene variants experimentally, there are programs to predict the significance of novel mutations in suspected genes. In this case, novel mutations were not found in the 100 normal healthy Chinese controls or in the 1000 human genome database. They were assumed to be detrimental to protein function by both SIFT and PolyPhen software analysis. Therefore, although we have not undertaken functional studies, we think the novel non-truncating mutations of the *LAMB2* gene were probably linked to the only renal phenotype in the present case. The implications of *NPHP1* mutations superimposed on *LAMB2* mutations are uncertain and require further observation in more cases.

Pregnancies carrying the foregoing deleterious gene mutations are undesirable due to pathogenic implications.

## Conclusion

Our case highlights the relationship between mutations in *LAMB2* and *NPHP1* in infants with isolated CNS. Next generation sequencing enables high-throughput screening of mutant genes rapidly, with clinically significant outcomes. In addition, our results expand the phenotype spectrum of only renal manifestations in disease associated with *LAMB2* mutation. Additional investigations are needed to determine the causal relationship between these gene mutations and disease. That mutations of *LAMB2* and *NPHP1* are occurred casually or probably associated still needs further investigation. Finally, urine tests are recommended for all edematous newborns to enable early detection of congenital nephrotic syndrome. Next generation sequencing facilitates screening of gene mutations in patients with congenital nephrotic syndrome.

### Consent

All examinations and investigations in this case were approved by the Ethical Committee of Tongji Hospital of Huazhong University of Science & Technology (China) and were conducted in accordance with the Declaration of Helsinki. Written informed consent was obtained from the parents for publication of this case report and accompanying images. A copy of the written consent is available for review by the Editor of *BMC Pediatrics*.
